# The Effects of Movement-Based Exercises Combined with Respiratory Techniques on the Thoracic Kyphosis Angle in Individuals with Thoracic Hyperkyphosis: A Systematic Review and Meta-Analysis

**DOI:** 10.3390/healthcare14152384

**Published:** 2026-08-04

**Authors:** Fatih Kirkbir, Hossein Khazanin, Mohammad Delavari, Shadi Moazamigoudarzi, Fatih Yilmaz, Izzet Karakulak, Oguz Yilmaz, Mojtaba Khosravi, Mohammad Alimoradi, Mohammad Alghosi, Monica Solana-Tramunt

**Affiliations:** 1 Department of Nutrition and Dietetics, Faculty of Health Sciences, Black Sea Technical University, Trabzon 61080, Turkey; fatihkirkbir@ktu.edu.tr; 2 Department of Sports Injury and Corrective Exercise, Faculty of Physical Education and Sport Sciences, University of Guilan, Rasht 4199613776, Iran; hosseinkhazanin@gmail.com (H.K.); delavari90mohammad@gmail.com (M.D.); 3 Department of Sciences for Quality of Life, University of Bologna, 40126 Rimini, Italy; shad.moazamigoudarzi@studio.unibo.it; 4 Department of Physical Education and Sports Sciences, Selçuk University, Konya 42130, Turkey; ftherenyilmaz@gmail.com; 5 Department of Sports Management, Faculty of Sport Sciences, Mardin Artuklu University, Mardin 47200, Turkey; izzetkarakulak@gmail.com; 6 Independent Researcher, Aydin 09100, Turkey; oguzyilmaz0626@gmail.com; 7 Department of Sports Medicine and Emerging Technologies, Faculty of Sport Sciences and Health, University of Tehran, Tehran 1417935840, Iran; mojtaba.khosravi.ce@gmail.com; 8 Department of Sports Injuries and Corrective Exercises, Faculty of Sports Sciences, Shahid Bahonar University of Kerman, Kerman 7616913439, Iran; malimoradisport@gmail.com; 9 HERC—Health, Exercise & Research Center, Mina Rashid, Dubai Maritime City, Dubai 50819, United Arab Emirates; 10 Department of Physical Education, Technical and Vocational University (TVU), Tehran 1435661137, Iran; mohammadalghosi9@gmail.com; 11 Department of Sport Sciences, Facultat en Ciències de l’Educació I l’Esport Blanquerna, Universitat Ramon Llull, 08022 Barcelona, Spain

**Keywords:** posture, corrective exercise, breathing exercises, postural balance, respiratory mechanics

## Abstract

**Highlights:**

**What are the main findings?**

- Combined movement-based and respiratory exercises significantly reduced thoracic kyphosis angle compared with exercise alone (moderate certainty evidence).
- The intervention demonstrated significant efficacy in adolescents (Hedges’ g = 0.860), indicating consistent and robust effects in younger populations.
- No significant effect was observed in older adults (Hedges’ g = 0.241), suggesting age-related differences in treatment response.

**What are the implications of the main findings?**

- Clinicians may consider adding respiratory techniques to postural correction programs, particularly for adolescents with thoracic hyperkyphosis.
- Routine assessment of breathing patterns and thoracic mobility is recommended in individuals with thoracic hyperkyphosis.
- Firm clinical recommendations await larger, high-quality randomized controlled trials, especially in older adult populations.

**Abstract:**

Background: Thoracic hyperkyphosis (THK) is a postural deformity associated with pain, impaired respiratory function, reduced quality of life, scapular rhythm impairment, shoulder biomechanical alterations, and increased fall risk. While corrective exercises are commonly used, the added value of integrating specific respiratory techniques remains unclear. This systematic review and meta-analysis aimed to evaluate the effects of movement-based exercises combined with respiratory techniques on the thoracic kyphosis angle (TKA) in individuals with THK. Methods: A systematic search of PubMed, Scopus, Web of Science, CINAHL, SPORTDiscus, PEDro, and CENTRAL Cochrane Library was conducted from inception to 16 July 2026. Randomized controlled trials (RCTs) and non-randomized controlled trials (non-RCTs) investigating combined exercise and respiratory interventions in individuals with THK were included. The primary outcome was TKA. Two authors independently selected studies, extracted data, and assessed quality, with a third author resolving discrepancies. Risk of bias was assessed using RoB-2 for RCTs and ROBINS-I for non-RCTs. Certainty of evidence was graded using GRADE. A random-effects meta-analysis of TKA was performed using Hedges’ g. Results: Five studies involving 247 participants were included. Meta-analysis demonstrated a significant reduction in TKA following combined intervention compared with exercise alone (Hedges’ g = 0.667, 95% CI: 0.395 to 0.940, *p* < 0.001; I^2^ = 39.7%). Subgroup analysis by age group revealed significant improvement in adolescents (Hedges’ g = 0.860, 95% CI: 0.532 to 1.189, *p* < 0.001; I^2^ = 0%) but no significant effect in older adults (Hedges’ g = 0.241, 95% CI: −0.247 to 0.729, *p* = 0.333; I^2^ = 0%). The certainty of evidence for TKA was graded as moderate, with downgrading for risk of bias, indirectness, and potential publication bias. Conclusions: Movement-based exercises combined with respiratory techniques significantly improve thoracic alignment in individuals with THK, with moderate certainty evidence, particularly in adolescents. The evidence in older adults remains inconclusive due to limited studies and structural factors. Larger, high-quality, and standardized trials are needed to confirm these effects, establish optimal treatment parameters, and evaluate long-term outcomes across diverse populations.

## 1. Introduction

Thoracic hyperkyphosis (THK) is characterized by an excessive anterior curvature of the thoracic spine as a postural condition [1]. The thoracic kyphosis angle (TKA) is an important indicator of THK severity, defined as angles exceeding 40° [2,3]. THK may be structural or postural. Structural hyperkyphosis results from fixed vertebral deformities, whereas postural hyperkyphosis is flexible and primarily related to muscular imbalance and poor posture [2]. THK is more prevalent among older individuals and tends to progress with advancing age, particularly in postmenopausal women [4]. Intensive engagement in gymnastics, swimming, cycling, and combat or overhead sports (e.g., tennis or handball) may elevate the risk of hyperkyphosis through cumulative repetitive loading, sport-specific muscular imbalances, and progressive structural adaptations of the immature spine. Additionally, poor postural habits, such as maintaining poor spinal alignment during daily activities, play a crucial role in the development of hyperkyphosis. This suggests that individuals across different groups may be at risk of developing hyperkyphosis due to lifestyle-related factors [5]. THK is associated with several impairments in the musculoskeletal, neuromuscular, and sensory systems, which may contribute to dysfunctions beyond the scapular complex, thoracic spine, and rib cage [6]. Increases in pain and risk of shoulder dysfunction, compromised function, and alignment in the cervical, thoracic, and lumbar spines are commonly observed in association with THK [4]. Biomechanical changes, including greater anterior movement of the center of gravity and increased postural sway [7,8,9], may impair balance maintenance ability and are associated with an increased risk of falling in THK [10]. Additionally, increased thoracic kyphosis is associated with respiratory complications, including poor respiratory function, dyspnea, and decreased Vital Capacity [11]. A greater TKA is associated with reduced pulmonary capacity, restricted chest wall expansion, and limited thoracic mobility [12,13]. This evidence suggests THK is a postural condition with multidimensional functional consequences, affecting quality of life and increasing mortality risk in affected individuals [14]. Therefore, investigating non-invasive approaches to either prevent or mitigate the consequences of THK is crucial.

Corrective exercise is an effective approach that improves thoracic spine mobility, stretches shortened anterior structures, strengthens weakened posterior chain muscles, and reduces the TKA [15]. Additionally, recent studies suggest that the combination of breathing techniques and exercise with a therapeutic emphasis not only improves postural deformity but also enhances respiratory function [16,17]. These effects may be mediated through improvements in diaphragmatic function, increased rib cage mobility, and enhanced activation of deep trunk stabilizing muscles [2]. Improved breathing mechanics may also facilitate thoracic extension, reduce chest wall stiffness, and enhance respiratory–postural coordination, thereby contributing to improved spinal alignment in individuals with THK [11,12,13]. Together, these mechanisms contribute to improved thoracic alignment, better postural control, and more efficient breathing mechanics in individuals with THK. However, while individual studies have reported improvements in THK, particularly reductions in the TKA, these findings should be interpreted with caution [16,18]. The TKA is a key outcome for diagnosing and managing THK. Available studies differ in their intervention protocols, population characteristics, and measurement methods. In addition, the methodological quality and risk of bias across trials should be carefully considered when interpreting the findings.

Therefore, a systematic review and meta-analysis are necessary to synthesize the available evidence and critically assess the quality of studies. This study will help determine the strength of the evidence and provide more reliable recommendations for clinical practice and future research. To the best of our knowledge, no systematic review and meta-analysis has yet evaluated the impact of combined movement-based and respiratory interventions on the TKA. Therefore, this study aims to evaluate and synthesize existing evidence on the effects of movement-based exercises combined with respiratory techniques on the TKA in individuals with THK.

## 2. Materials and Methods

### 2.1. Protocol Registration

This systematic review report adhered to the Preferred Reporting Items for Systematic Reviews and Meta-Analyses (PRISMA) guideline [19] (Supplementary Table S1). The review is registered in the International Prospective Register of Systematic Reviews (PROSPERO) under the registration number CRD420251275146, registered on 29 December 2025, and updated on 19 July 2026.

### 2.2. Protocol Deviations

Four deviations from the registered protocol were made. First, the search was expanded from the originally registered databases (MEDLINE, PubMed, SSCI, and Scopus) to include Web of Science, CINAHL, SPORTDiscus, PEDro, and CENTRAL Cochrane, and the search period was extended beyond the original timeline to 16 July 2026, ensuring comprehensive coverage and capture of newly published studies. Second, although the protocol stated that meta-analysis would not be performed due to anticipated heterogeneity, a meta-analysis was conducted for TKA, as five studies reported this outcome with sufficient data, enabling meaningful quantitative synthesis and more robust findings. Third, the inclusion criteria were refined to require that the comparator group receive the same movement-based exercise without the respiratory component, a methodological clarification that was not fully specified in the original protocol but was essential to isolate the added value of respiratory techniques. Fourth, subgroup analysis by age group (adolescents vs. older adults) was not pre-specified but was added post hoc to explore substantial clinical heterogeneity observed during data synthesis. No other deviations occurred.

### 2.3. Search Strategy

A systematic search was conducted across the Web of Science, PubMed, Scopus, CINHAL, SPORTDiscus, PEDro, and the CENTRAL Cochrane Library from their inception through 16 July 2026. The search strategies followed the PRESS (Peer Review of Electronic Search Strategies) guideline statement [20]. The search strategy incorporated MeSH terms, Emtree terms, and text words, combining multiple entry strings with Boolean operators (AND/OR) to develop a detailed search syntax for the two main concepts: kyphosis and exercise interventions. No language or date restrictions were applied. Table 1 presents the complete search strategies for each database. To supplement the electronic searches, secondary searches were conducted: (a) screening the reference lists of all included studies and relevant review papers; (b) examining studies that cited the included studies (forward citation tracking) via Google Scholar, screening the first 200 results (Table 1); and (c) Using Connected Papers (https://www.connectedpapers.com, accessed on 16 July 2026), key included studies identified during the initial database searches were used as seed papers to identify additional relevant studies through visual citation mapping. Two authors (H.K. and M.K.) independently carried out the searches, resolving any discrepancies through discussion or, if needed, consulting a third author (F.K.).

### 2.4. Study Selection

The inclusion and exclusion criteria for the studies were established using the PICOS (Population, Intervention, Comparison, Outcome, and Study design) framework [21], as outlined in Table 2. Two authors (H.K. and M.K.) independently screened the titles and abstracts of all identified records against the eligibility criteria. Potentially relevant full-text articles were then retrieved and independently assessed for eligibility by the same two authors (H.K. and M.K.). Any disagreements during the screening process were resolved through discussion; if consensus could not be reached, a third author (F.K.) was consulted to arbitrate. Any non-English articles identified would have been translated using Google Translate and verified by a language-proficient reviewer.

### 2.5. Data Extraction

Data from the included studies were independently collected by two authors (M.D. and S.M.), and study details such as author, publication year, and location, as well as study design, participant information (including sample size, sex, and age), measurement tool, baseline kyphosis value, descriptions of the interventions, outcomes, and results were included. For missing or inconsistent data, the authors were contacted via email (up to 3 attempts, spaced 72 h apart) and, when applicable, via ResearchGate. If no reply was received after three attempts or if the necessary information was not provided, the relevant study outcome was excluded from further analysis (n = 0).

### 2.6. Quality Assessment

The evaluation of risk of bias within the included studies was performed in accordance with Cochrane methodological standards. The ROBINS-I instrument was applied to assess bias in non-randomized controlled trials (non-RCTs) [23], whereas the RoB-2 tool was utilized for randomized controlled trials (RCTs) [24]. Two independent reviewers (F.Y. and I.K.) systematically evaluated each study by reviewing all relevant domains; any disagreements were resolved by a third author (O.Y.). For non-RCTs, the seven core ROBINS-I domains, confounding, participant selection, intervention classification, deviations from intended interventions, missing outcome data, outcome measurement, and reporting selection were examined. For RCTs, the five RoB-2 domains were assessed, with particular attention to potential bias arising from the randomization process.

Both tools offered structured guidance supported by signaling questions, enabling consistent and transparent judgments across studies. Ratings included classifications such as “low risk of bias”, “high risk of bias”, “some concerns”, “moderate”, and “unclear risk” or “no information” supplemented by descriptive rationale for each judgment.

### 2.7. Data Synthesis

Prior to data integration, inter-reviewer agreement at each stage of the review process (title/abstract screening, full-text screening, and methodological quality assessment) was systematically assessed using Kappa (κ) statistics. Agreement was categorized as poor (κ ≤ 0.20), fair (κ = 0.21–0.40), moderate (κ = 0.41–0.60), substantial (κ = 0.61–0.80), or near-perfect (κ = 0.81–0.99) [25].

A quantitative meta-analysis was performed for TKA, the only outcome reported by all five included studies, whereas a meta-analysis was not feasible for the other outcomes, each reported by only one study. The effect measure was calculated as the standardized mean difference (Hedges’ g) between the intervention group (movement-based exercise combined with respiratory techniques) and the control group (movement-based exercise alone), using post-intervention means or change scores, with correction for small-sample bias. A random-effects model (DerSimonian-Laird method) was employed for all pooled estimates to account for anticipated clinical and methodological heterogeneity. Heterogeneity was quantified using the I^2^ statistic and assessed with Cochran’s Q test; I^2^ values > 50% were considered substantial. Publication bias was explored using visual inspection of the funnel plot, supplemented by Egger’s linear regression test to assess funnel plot asymmetry [26]. However, tests for funnel plot asymmetry have limited statistical power when fewer than 10 studies are included (Cochrane Handbook) [27], and a non-significant result does not exclude the possibility of publication bias or other small-study effects (n = 5). Therefore, these results should be interpreted with considerable caution. Pre-planned subgroup analyses were conducted by age group based on clinical rationale: adolescents (<18 years; 3 studies) and older adults (≥50 years; 2 studies). A leave-one-out sensitivity analysis was performed for each outcome to assess the robustness of the pooled estimates, sequentially removing one study at a time and recalculating the summary effect size. All statistical tests were two-sided, with significance set at *p* < 0.05. Analyses were performed using Comprehensive Meta-Analysis software version 4 (CMA; Biostat, Englewood, NJ, USA).

### 2.8. Grading of Evidence Quality

The certainty of evidence for each primary outcome was evaluated using the Grading of Recommendations, Assessment, Development, and Evaluation (GRADE) system [28], as outlined in the Cochrane Handbook for Systematic Reviews of Interventions [29]. This framework assesses evidence across five domains: risk of bias, inconsistency, indirectness, imprecision, and publication bias. Evidence certainty is classified as high, moderate, low, or very low [28]. For TKA, the initial certainty level was set to high for RCTs (3 of 5 studies) and to low for non-RCTs (2 of 5 studies), in accordance with GRADE guidance. This framework evaluates evidence across five domains: (i) risk of bias, (ii) imprecision, (iii) inconsistency, (iv) indirectness, and (v) publication bias. The certainty of evidence is reduced by one level if any of the following issues arise: moderate or high risk of bias, effect estimates with confidence intervals that cross the null, significant heterogeneity (I^2^ > 50%), an indirect outcome measurement, or indications of potential or unexplained publication bias. The certainty of evidence ratings was independently assessed by two reviewers (H.K. and M.K.), with disagreements resolved through discussion or consultation with a third author (M.ALI.).

## 3. Results

### 3.1. Study Identification

**Figure 1:**
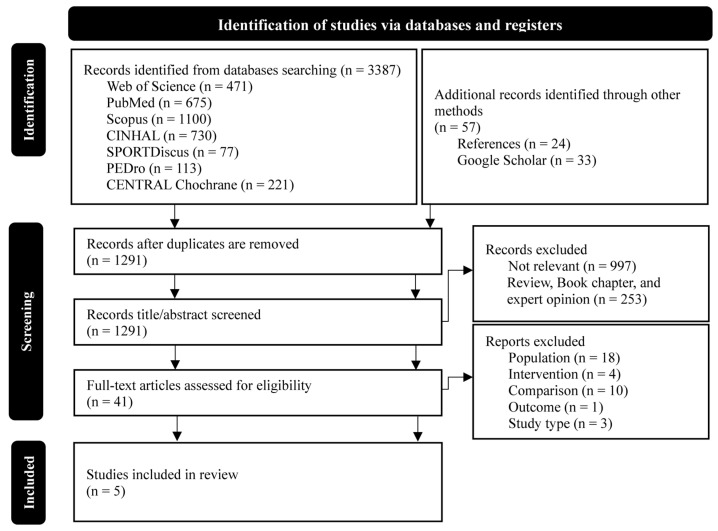
Preferred Reporting Items for Systematic Reviews and Meta-Analyses (PRISMA) 2020 flow diagram for new systematic reviews, including searches of databases and registers.

An initial search of electronic databases identified a total of 3387 records: Web of Science (471; 13.91%), PubMed (675; 19.93%), Scopus (1100; 32.48%), CINAHL (730; 21.55%), SPORTDiscus (77; 2.27%), PEDro (113; 3.34%), and CENTRAL Cochrane (221; 6.52%). After duplicate removal, 1291 records remained. A Google Scholar search retrieved 24 additional records, and screening the reference lists of relevant studies identified 24 further records. During title and abstract screening, 1250 records were excluded, leaving 41 articles for full-text assessment. Following a thorough evaluation of the full texts and the application of predefined inclusion and exclusion criteria, 5 studies were deemed eligible and included in this systematic review and meta-analysis, while 36 studies were excluded, as detailed in Supplementary Table S2. Inter-reviewer agreement was near-perfect at both stages of screening, with κ = 0.87 for title and abstract screening and κ = 0.91 for full-text screening. The included studies examined the effects of movement-based exercises combined with respiratory exercises on TKA. This systematic review and meta-analysis aimed to evaluate the impact of these interventions on the aforementioned outcome. The flow of studies through each screening stage is summarized in the PRISMA diagram (Figure 1).

### 3.2. Descriptive Characteristics of the Included Studies

The five studies included in this systematic review and meta-analysis, published between 2020 and 2025, encompassed a total of 247 participants. The sample sizes ranged from 22 to 118 participants. Out of 247 participants, 67 (27.1%) were male, and 180 (72.9%) were female. Three studies included only female participants [17,18,30], while two studies included only male participants [31,32]. The included studies were conducted across three countries: Egypt (n = 2), Iran (n = 2), and Indonesia (n = 1). Among the five studies [17,18,30,31,32], three were RCTs conducted in Iran (n = 1) and Egypt (n = 2), while two were non-RCTs conducted in Indonesia (n = 1) and Iran (n = 1). In terms of age distribution, the studies comprised two distinct subgroups: adolescents (three studies; mean age range: 13.0–14.0 years) and older adults (two studies; mean age range: 56.4–59.9 years). This bimodal age distribution reflects the clinical heterogeneity between developmental and age-related hyperkyphosis. The baseline TKA ranged from 48.7° to 62.2° across studies, with measurement tools including a flexible ruler (n = 3), Cobb’s method on standing lateral spine X-ray (n = 1), and a kyphometer (n = 1). Four of the five included studies [17,30,31,32] reported that exercise sessions were supervised by a physical therapist or trained instructor. Attrition was reported in only one study [17], with a dropout rate of 15.3% (18 of 118 participants). No study reported intervention adherence rates (e.g., attendance or compliance) or adverse events. Intervention fidelity was not reported in any of the included studies. A detailed overview of the characteristics of the included studies is presented in Table 3.

### 3.3. Evaluation of Risk of Bias with ROB-2 and ROBINS-I

The methodological quality of the five included studies was assessed using the RoB-2 tool for RCTs (n = 3) and the ROBINS-I tool for non-RCTs (n = 2). Inter-rater agreement was near-perfect (κ = 0.89), confirming consistent bias assessments across reviewers.

Based on the overall RoB-2 assessment, all three RCTs were rated as having some concerns [17,30,32]. Domain-specific assessments for the RCTs were as follows: during randomization, all three studies raised concerns due to insufficient detail regarding allocation concealment. In deviations from intended interventions, all three studies were rated as low risk. For missing outcome data, two studies were rated as low risk, while one study raised some concerns. In the measurement of the outcome, two studies were rated as low risk, while one study raised some concerns. In selecting the reported results, all three studies were rated as low risk.

Among the two non-RCTs, one study [18] was rated as having a serious overall risk of bias, while the other [31] was rated as having a moderate overall risk of bias. Domain-specific assessments using ROBINS-I showed that, in the confounding domain, one study was rated as serious and one moderate. In the participant selection section, both studies were rated as low risk. In the classification of interventions, both studies were rated as low risk. In deviations from intended interventions, both studies were rated as low risk. For missing data, both studies were rated as moderate risk. In outcome measurement, both studies were rated as moderate risk. In the selection of the reported results, both studies (100%) were rated as low risk.

**Figure 2:**
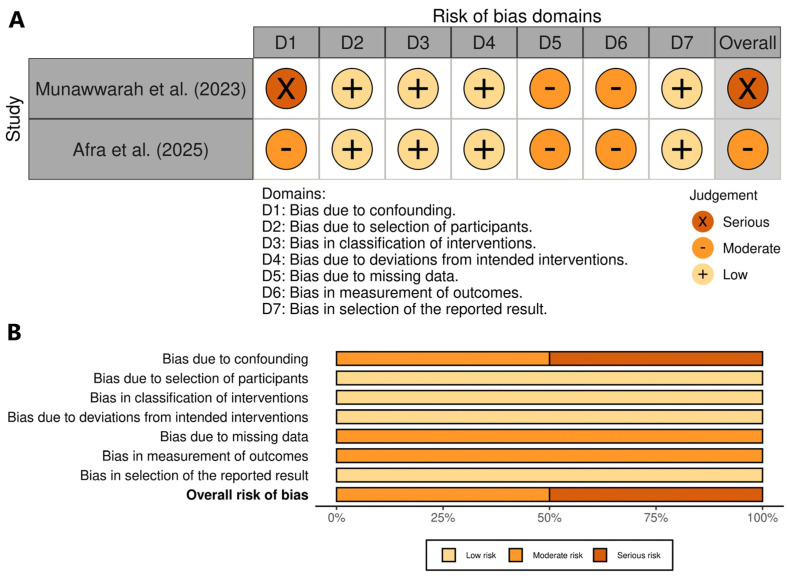
Summary of risk of bias among the included non-RCTs [18,31] assessed via ROBINS-I: (**A**) traffic light plot; (**B**) summary plot.

**Figure 3:**
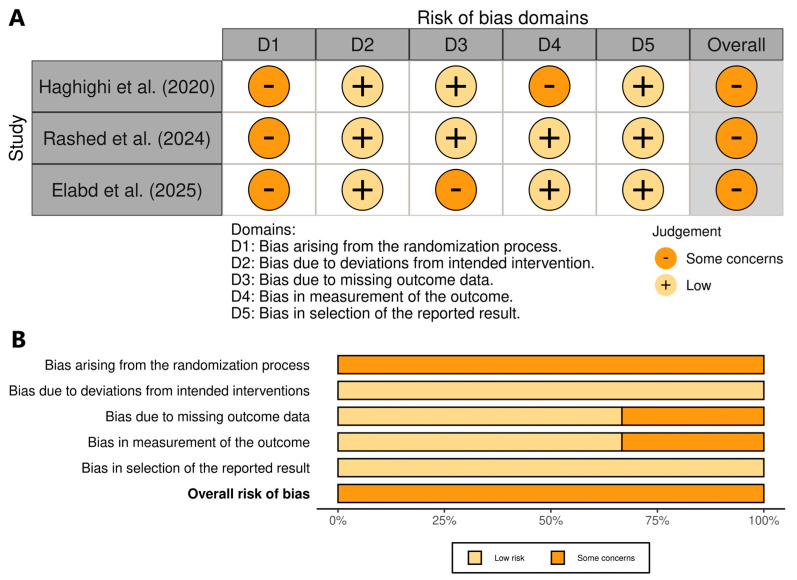
Summary of risk of bias among the included RCTs [17,30,32] assessed via RoB-2: (**A**) traffic light plot; (**B**) summary plot.

Overall, across the seven domains of the ROBINS-I tool for the two non-RCTs, eight domains (57%) were rated as low risk, five domains (36%) as moderate risk, and one domain (7%) as serious risk. A detailed summary of the risk-of-bias assessment is presented in Figure 2 and Figure 3, including the traffic-light plot (A) and the overall risk-of-bias summary plot (B) (www.riskofbias.info, accessed on 18 July 2026) [33].

### 3.4. Meta-Analysis of Thoracic Kyphosis Angle

A random-effects meta-analysis was performed on five studies that provided sufficient data for pooling to evaluate the effects of movement-based exercises combined with respiratory techniques on TKA. The pooled analysis demonstrated a statistically significant reduction in TKA following the combined intervention compared with movement-based exercise alone (Hedges’ g = 0.667, 95% CI: 0.395 to 0.940, *p* < 0.001), with low heterogeneity observed across studies (I^2^ = 39.7%), indicating moderate consistency among the findings.

**Figure 4:**
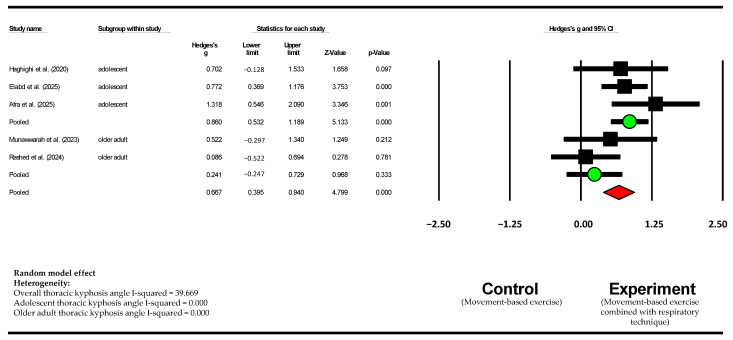
Forest plot of thoracic kyphosis angle movement-based exercise combined with respiratory technique vs. movement-based exercise alone [17,18,30,31,32]. Green circles indicate the pooled effect sizes for each subgroup (adolescents and older adults). The red diamond indicates the overall pooled effect size.

Subgroup analysis by age group was performed to explore whether intervention effects differed between adolescents and older adults. In the adolescent subgroup (three studies [17,31,32]), the combined intervention produced a statistically significant improvement (Hedges’ g = 0.860, 95% CI: 0.532 to 1.189, *p* < 0.001; I^2^ = 0%). In contrast, the older adult subgroup (two studies; [18,30]) did not demonstrate a statistically significant effect (Hedges’ g = 0.241, 95% CI: −0.247 to 0.729, *p* = 0.333; I^2^ = 0%). Figure 4 presents the forest plot for the overall analysis and subgroup analyses by age group.

### 3.5. Publication Bias

**Figure 5:**
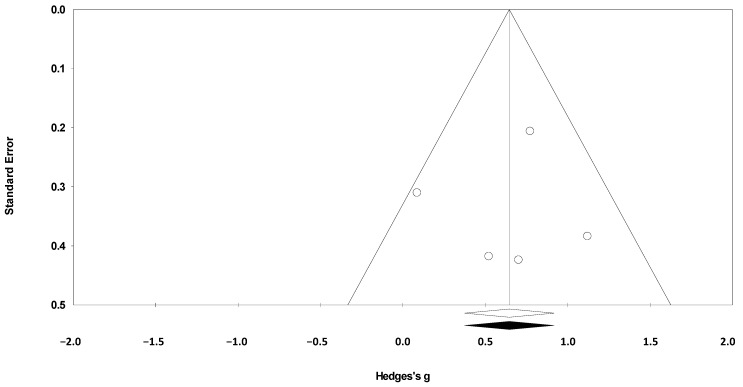
Funnel plot of thoracic kyphosis angle meta-analysis.

Publication bias was assessed using Egger’s linear regression test, supplemented by visual inspection of the funnel plot. Egger’s test did not reveal statistically significant evidence of funnel plot asymmetry (intercept = 0.058, SE = 2.263, 95% CI: −7.145 to 7.261, *p* = 0.981). Visual inspection of the funnel plot suggested an approximately symmetrical distribution of studies, with no clear indication of publication bias (Figure 5). However, given the small number of included studies (n = 5), these results should be interpreted with considerable caution, as the tests have limited power and cannot rule out publication bias.

### 3.6. Sensitivity Analysis

A leave-one-out sensitivity analysis was performed to assess the robustness of the pooled effect estimate for TKA. The overall pooled effect size was Hedges’ g = 0.667 (95% CI: 0.395 to 0.940; *p* < 0.001). Sequentially removing each study revealed that the effect remained statistically significant across all analyses, with effect sizes ranging from 0.557 (95% CI: 0.230 to 0.885; *p* = 0.001) after removing Afra et al. (2025) [31] to 0.813 (95% CI: 0.508 to 1.118; *p* < 0.001) after removing Rashed et al. (2024) [30]. These findings confirm that the overall TKA result was not unduly influenced by any single study and is robust. Detailed sensitivity analysis results are presented in Table 4.

### 3.7. Certainty of the Evidence

The certainty of evidence for TKA was assessed using the GRADE system [28]. Evidence certainty was downgraded by one level for risk of bias, as all three RCTs had “some concerns” (RoB-2) and both non-RCTs had “serious” or “moderate” risk (ROBINS-I). No downgrade was applied for inconsistency, as heterogeneity was low (I^2^ = 39.7%) with consistent effect direction. Indirectness led to downgrading due to distinct age subgroups (adolescents vs. older adults) and varied measurement tools. Imprecision did not warrant downgrading, given the narrow confidence interval and sufficient sample size (n = 247). Potential publication bias contributed to downgrading, as it could not be reliably excluded with only five studies. Overall, the certainty of evidence for TKA was rated as moderate (⨁⨁⨁◯), indicating moderate confidence that the true effect is close to the estimated effect (Table 5).

## 4. Discussion

The present systematic review and meta-analysis synthesized evidence from five studies involving 247 participants to evaluate the effects of movement-based exercises combined with respiratory techniques on TKA in individuals with THK. The pooled analysis demonstrated a statistically significant reduction in TKA following the combined intervention compared with movement-based exercise alone (Hedges’ g = 0.667, 95% CI: 0.395 to 0.940, *p* < 0.001), with low heterogeneity across studies (I^2^ = 39.7%). Subgroup analyses revealed that the intervention was highly effective in adolescents (Hedges’ g = 0.860, 95% CI: 0.532 to 1.189, *p* < 0.001; I^2^ = 0%), whereas no statistically significant effect was observed in older adults (Hedges’ g = 0.241, 95% CI: −0.247 to 0.729, *p* = 0.333; I^2^ = 0%). These findings suggest that adding respiratory techniques to movement-based exercise may improve thoracic alignment, particularly in younger populations.

The present results align with the broader body of evidence supporting exercise interventions for THK. Jenkins et al. conducted a systematic review and meta-analysis of 28 studies and found that structured exercise programs of three months’ duration or less were effective in reducing THK in both younger (SMD −2.8, 95% CI −4.3 to −1.3) and older populations (SMD −0.3, 95% CI −0.6 to 0.0), with low to moderate-quality evidence [34]. The effect sizes observed in the present review for adolescents (Hedges’ g = 0.860) are comparable to those reported by González-Gálvez et al., who found a large, significant effect of exercise on kyphosis angle (SMD = −1.400, 95% CI −2.150 to −0.660, *p* = 0.000) [35]. Similarly, Fernández et al. reviewed nine RCTs and concluded that therapeutic exercise appears effective in managing hyperkyphosis in adolescents and young adults [36]. The consistency of these findings across independent reviews supports the conclusion that exercise-based interventions improve thoracic posture.

Several prior reviews have focused exclusively on older adult populations, providing a useful contrast to the present findings. Kumar and Singla conducted a systematic review of 8 studies involving 463 hyperkyphotic older adults and concluded that exercise is effective in improving posture; however, they were unable to estimate the effects of exercise on pulmonary function, gait, or quality of life due to limited and inadequate data [37]. Ponzano et al. performed a meta-analysis of 24 studies in adults aged 45 years and older with hyperkyphosis and found that exercise improved kyphosis outcomes with moderate certainty evidence (SMD −0.31, 95% CI −0.46 to −0.16) [38]. The smaller effect size observed in older adult populations (SMD −0.31) compared with the present adolescent subgroup (Hedges’ g = 0.860) likely reflects the more complex etiology of age-related hyperkyphosis, which involves vertebral compression fractures, disc degeneration, and osteoporosis, factors that may be less responsive to exercise interventions alone [4]. Indeed, the present subgroup analysis found no statistically significant effect in older adults (Hedges’ g = 0.241, 95% CI −0.247 to 0.729, *p* = 0.333), a finding that may be attributable to the small number of studies in this subgroup (n = 2) as well as the multifactorial nature of age-related hyperkyphosis.

The present review extends previous work by specifically examining the added value of respiratory techniques when combined with movement-based exercise. While previous systematic reviews have evaluated exercise interventions for hyperkyphosis, few have isolated the respiratory component as a distinct therapeutic element. The physiological rationale for this approach is well established: THK restricts rib-cage expansion, impairs diaphragmatic function, and reduces pulmonary capacity [39]. By integrating respiratory techniques, such as diaphragmatic breathing, inspiratory muscle training, and respiratory muscle training, the combined intervention may address both the biomechanical and respiratory consequences of hyperkyphosis. The present findings suggest that this dual approach yields a moderate-to-large effect in adolescents, potentially because younger individuals have greater spinal flexibility and neuromuscular plasticity, allowing them to derive greater benefit from respiratory-motor integration. Furthermore, the low heterogeneity observed in the adolescent subgroup indicates that the intervention effect is consistent across studies, enhancing confidence in these findings.

The observed age-related difference in treatment response warrants careful consideration. Adolescents with THK typically present with postural or flexible kyphosis, which is amenable to corrective exercise and neuromuscular re-education [36,40]. In contrast, older adults often present with structural hyperkyphosis resulting from vertebral compression fractures, disc degeneration, and osteoporosis [1,4]. These structural changes may limit the extent to which exercise, even when combined with respiratory techniques, can modify the kyphotic angle. Additionally, the present meta-analysis included only two studies in older adults [18,30], both of which used flexible ruler measurements rather than the gold standard radiographic Cobb angle. This methodological limitation may have contributed to the non-significant finding in this subgroup, as non-radiographic measures are less precise and may not detect small but clinically meaningful changes. The lack of statistical significance in older adults should therefore be interpreted with caution and does not necessarily indicate that the intervention is ineffective in this population.

### 4.1. Limitations

Several limitations of the present review should be acknowledged. First, the small number of included studies (n = 5) limits the meta-analysis’s statistical power and precludes a reliable assessment of publication bias. Although Egger’s test did not indicate significant funnel plot asymmetry (*p* = 0.981), this finding should be interpreted with considerable caution, given the limited number of studies. Second, the included studies exhibited considerable clinical heterogeneity across age groups (adolescents vs. older adults), measurement tools (flexible ruler, Cobb angle, kyphometer), and intervention protocols (varying duration, frequency, and types of respiratory techniques). While random-effects models were used to account for this heterogeneity, the diversity of interventions limits the ability to draw definitive conclusions about optimal treatment parameters. Third, the present review was unable to perform meta-analyses for pulmonary function outcomes or craniovertebral angle, as these were reported by only one study each. This represents a significant gap in the evidence base, as respiratory function is a key clinical outcome in individuals with THK.

### 4.2. Clinical Implications

The findings of this systematic review and meta-analysis have several important implications for clinical practice and research. For clinicians, moderate-certainty evidence supports the use of combined movement-based and respiratory exercise programs to improve thoracic alignment in adolescents with hyperkyphosis. The addition of respiratory techniques, such as diaphragmatic breathing, inspiratory muscle training, and respiratory muscle training, appears to provide added benefit beyond movement-based exercise alone, particularly in younger populations. Clinicians should consider assessing respiratory function and incorporating breathing exercises into postural correction programs, especially for adolescents with THK. For older adults, the evidence is less conclusive, and clinicians should be cautious in recommending this intervention as a standalone treatment for age-related hyperkyphosis, given the structural and degenerative factors that may limit responsiveness.

From a research perspective, the present findings highlight the need for high-quality RCTs with adequate sample sizes, standardized outcome measures, and long-term follow-up. Future studies should use the gold standard radiographic Cobb angle measurement to ensure comparability across studies and should report detailed information on intervention parameters (duration, frequency, intensity, and type of respiratory techniques) to facilitate replication and meta-analysis. The absence of pooled estimates for pulmonary function outcomes underscores the need for future trials to include comprehensive respiratory assessments.

### 4.3. Future Research Directions

Several directions for future research emerge from the present review. First, large-scale, high-quality RCTs are urgently needed to confirm the efficacy of combined movement-based and respiratory exercise interventions for THK, particularly in older adult populations where the evidence is currently insufficient. Second, future studies should employ standardized outcome measures, including radiographic Cobb angle, pulmonary function tests, and patient-reported outcomes such as quality of life and pain, to enable meaningful comparisons across studies. Third, research should investigate the optimal dosage and duration of respiratory techniques, specifically, whether certain types of breathing exercises (e.g., diaphragmatic breathing vs. inspiratory muscle training) confer greater benefit and whether the effects are sustained beyond the intervention period. Fourth, mechanistic studies are needed to elucidate the physiological pathways through which respiratory techniques influence thoracic alignment, for example, whether improvements in diaphragmatic function and rib cage mobility mediate reductions in the kyphotic angle. Fifth, given the age-related differences observed in the present subgroup analyses, future research should stratify participants by age and etiology of hyperkyphosis (postural vs. structural) to better understand which populations are most likely to benefit from combined interventions. Finally, the lack of evidence for pulmonary function outcomes should be addressed through dedicated trials that include comprehensive respiratory assessments.

## 5. Conclusions

This systematic review and meta-analysis provide moderate-certainty evidence that movement-based exercises combined with respiratory techniques significantly reduce the TKA compared with movement-based exercises alone. The intervention demonstrates particular efficacy in adolescents, whereas the evidence in older adults remains inconclusive, likely due to the limited number of studies and the structural nature of age-related hyperkyphosis. The findings support integrating respiratory techniques into postural correction programs, especially for younger populations, and highlight the need for high-quality RCTs to confirm these effects, establish optimal treatment parameters, and evaluate long-term outcomes. Despite the limitations of the current evidence base, including small sample sizes, methodological concerns, and heterogeneity in outcome measures, the present review contributes to the growing body of literature supporting exercise-based interventions for THK and underscores the potential added value of respiratory training in improving spinal alignment and respiratory function.

## Figures and Tables

**Table 1:** Search queries for each database.

Database	Complete Search Strategy
Web of Science	(TS=(kyphosis OR hyperkyphosis OR “thoracic kyphosis” OR “flexed posture” OR “dowager’s hump” OR hunchback)) AND (TS=(“breathing exercise” OR “respiratory training” OR “diaphragmatic breathing” OR “diaphragmatic breathing exercise” OR “respiratory muscle training” OR “inspiratory muscle training” OR “ventilatory muscle training” OR “respiratory exercise” OR “corrective exercise” OR “postural exercise” OR “exercise therapy” OR rehabilitation OR “thoracic mobility”))
PubMed	((“Kyphosis”[MeSH]) OR “Hyperkyphosis”[MeSH] OR kyphosis[Title/Abstract] OR hyperkyphosis[Title/Abstract] OR “thoracic kyphosis”[Title/Abstract] OR “flexed posture”[Title/Abstract] OR “dowager’s hump”[Title/Abstract] OR hunchback[Title/Abstract]) AND ((“Breathing Exercises”[MeSH]) OR “Respiratory Therapy”[MeSH] OR “Exercise Therapy”[MeSH] OR “breathing exercise”[Title/Abstract] OR “respiratory training”[Title/Abstract] OR “diaphragmatic breathing”[Title/Abstract] OR “respiratory muscle training”[Title/Abstract] OR “inspiratory muscle training”[Title/Abstract] OR “ventilatory muscle training”[Title/Abstract] OR “respiratory exercise”[Title/Abstract] OR “corrective exercise”[Title/Abstract] OR “postural exercise”[Title/Abstract] OR “thoracic mobility”[Title/Abstract] OR rehabilitation[Title/Abstract])
Scopus	(TITLE-ABS-KEY (kyphos* OR “thoracic kyphosis” OR “flexed posture” OR “dowager hump” OR hunchback)) AND (TITLE-ABS-KEY (“breathing exercise” OR “respiratory training” OR “diaphragmatic breathing” OR “respiratory muscle training” OR “inspiratory muscle training” OR “ventilatory muscle training” OR “respiratory exercise” OR “corrective exercise” OR “postural exercise” OR “exercise therapy” OR rehabilitation OR “thoracic mobility”))
CINHAL	(MH “Kyphosis”) OR (TI (kyphosis OR hyperkyphosis OR “thoracic kyphosis” OR “flexed posture” OR “dowager’s hump” OR hunchback) OR AB (kyphosis OR hyperkyphosis OR “thoracic kyphosis” OR “flexed posture” OR “dowager’s hump” OR hunchback)) AND (MH “Breathing Exercises”) OR (MH “Respiratory Therapy”) OR (MH “Exercise Therapy”) OR (TI (“breathing exercise” OR “respiratory training” OR “diaphragmatic breathing” OR “respiratory muscle training” OR “inspiratory muscle training” OR “ventilatory muscle training” OR “respiratory exercise” OR “corrective exercise” OR “postural exercise” OR “exercise therapy” OR rehabilitation OR “thoracic mobility”) OR AB (“breathing exercise” OR “respiratory training” OR “diaphragmatic breathing” OR “respiratory muscle training” OR “inspiratory muscle training” OR “ventilatory muscle training” OR “respiratory exercise” OR “corrective exercise” OR “postural exercise” OR “exercise therapy” OR rehabilitation OR “thoracic mobility”))
SPORTDiscus	(TI (kyphosis OR hyperkyphosis OR “thoracic kyphosis” OR “flexed posture” OR “dowager’s hump” OR hunchback) OR AB (kyphosis OR hyperkyphosis OR “thoracic kyphosis” OR “flexed posture” OR “dowager’s hump” OR hunchback)) AND (TI (“breathing exercise” OR “respiratory training” OR “diaphragmatic breathing” OR “respiratory muscle training” OR “inspiratory muscle training” OR “ventilatory muscle training” OR “respiratory exercise” OR “corrective exercise” OR “postural exercise” OR “exercise therapy” OR rehabilitation OR “thoracic mobility”) OR AB (“breathing exercise” OR “respiratory training” OR “diaphragmatic breathing” OR “respiratory muscle training” OR “inspiratory muscle training” OR “ventilatory muscle training” OR “respiratory exercise” OR “corrective exercise” OR “postural exercise” OR “exercise therapy” OR rehabilitation OR “thoracic mobility”))
PEDro	1. Abstract & Title: kyphosis AND breathing: 4 2. Abstract & Title: kyphosis AND respiratory: 8 3. Abstract & Title: kyphosis AND inspiratory: 4 4. Abstract & Title: kyphosis AND exercise: 71 5. Abstract & Title: kyphosis AND corrective: 26 6. Abstract & Title: kyphosis AND rehabilitation: 10
CENTRAL Cochrane	#1 MeSH descriptor: [Kyphosis] explode all trees #2 kyphosis OR hyperkyphosis OR “thoracic kyphosis” OR “flexed posture” OR “dowager’s hump” OR hunchback #3 #1 OR #2 #4 MeSH descriptor: [Breathing Exercises] explode all trees #5 MeSH descriptor: [Respiratory Therapy] explode all trees #6 MeSH descriptor: [Exercise Therapy] explode all trees #7 “breathing exercise” OR “respiratory training” OR “diaphragmatic breathing” OR “respiratory muscle training” OR “inspiratory muscle training” OR “ventilatory muscle training” OR “respiratory exercise” OR “corrective exercise” OR “postural exercise” OR rehabilitation OR “thoracic mobility” #8 #4 OR #5 OR #6 OR #7 #9 #3 AND #8
Google Scholar	(kyphosis OR hyperkyphosis OR “thoracic kyphosis”) AND (“breathing exercise” OR “respiratory training” OR “corrective exercise” OR rehabilitation)

**Table 2:** Eligibility of the studies based on the PICOS framework.

	Inclusion Criteria	Exclusion Criteria
Population	Individuals with thoracic hyperkyphosis, defined as a thoracic curvature ≥ 40° measured by radiographic or validated non-radiological methods. Radiographic measurement (gold standard) is defined as a Cobb angle ≥ 40° measured on a standing lateral thoracolumbar radiograph using Cobb’s method [4]. Non-radiological clinical measures are defined as thoracic curvature exceeding validated cut-off values, Cobb angle ≥ 40°, measured by Debrunner kyphometer (angle ≥ 40°), flexicurve kyphosis angle (angle ≥ 31.4°), flexicurve kyphosis index (index ≥ 0.115), flexible ruler, or inclinometer [22].	Secondary or pathological kyphosis (e.g., Scheuermann’s disease, vertebral fracture, tumor, trauma) or other structural spinal deformities (e.g., scoliosis) that may alter curvature and affect posture assessment Neuromuscular or systemic disorders impacting posture (e.g., Parkinson’s disease, multiple sclerosis, rheumatoid arthritis) History of spinal surgery or a recent vertebral fracture (<6 months) Cognitive or severe physical impairments that limit safe participation in corrective exercise Institutionalized adults
Intervention	Movement-based exercise programs that incorporate respiratory techniques, such as diaphragmatic breathing, respiratory re-education, deep breathing, inspiratory muscle training, resisted breathing, or dynamic neuromuscular stabilization-based breathing, aim to reduce the thoracic hyperkyphosis angle and improve overall posture.	Interventions lacking a respiratory component or focusing mainly on other modalities (e.g., yoga, Pilates, pharmacological therapy) without a clear breathing-based approach.
Comparison	Studies in which the intervention group received movement-based exercises combined with respiratory techniques, and the control group received the same movement-based exercise protocol without the respiratory component (i.e., matched active comparator).	Studies were excluded if: (a) all groups received respiratory techniques; (b) the control group received no intervention, usual care, walking, or a structurally different exercise protocol; (c) the study lacked a comparator group; or (d) the comparator was pharmacological or non-exercise-based.
Outcome	Thoracic kyphosis angle (in degrees) measured by validated tools, including Cobb angle (radiography), flexicurve, kyphometer, or inclinometer.	Studies not reporting quantitative kyphosis angle or using non-validated measurement tools.
Study design	Interventional studies: randomized controlled trials, quasi-experimental designs (e.g., nonrandomized controlled), and clinical trials Quantitative, original research reporting primary data	Observational studies Study protocols Case reports or case series Qualitative designs Non-interventional studies Non-original publications (e.g., reviews, meta-analyses, editorials, letters, commentaries, book chapters)

**Table 3:** Characteristics of the included studies.

Study Details	Study Design	Sample Description	Measurement Tool and Baseline Kyphosis Value (°)	Exercise Characteristics	Control Group Intervention	Outcomes	Results
Haghighi et al. (2020) Iran [32]	RCT	N: 22 Sex: male Age: 14.0 ± 1.6 years	Flexible ruler Baseline kyphosis value: 55.9	D: 12 weeks F: 3 times per week I: NR Ti: 60 min T: combined inspiratory muscle training with corrective exercise	D: 12 weeks F: 3 times per week I: NR T: 60 min T: corrective exercise	TKA CIE	A significant reduction in TKA in the inspiratory muscle training group compared with the control group (*p* = 0.025).
Munawwarah et al. (2023) Indonesia [18]	Non- RCT	N: 22 Sex: female Age: 59.9 ± 3.3 years	Flexible ruler Baseline kyphosis value: 54.4	D: 5 weeks F: NR I: NR Ti: NR T: combined deep breathing exercise with postural training	D: 5 weeks F: NR I: NR Ti: NR T: postural training	TKA	The changes in TKA were statistically significant (*p* = 0.000), and the between-group differences were likewise statistically significant (*p* < 0.001).
Rashed et al., (2024) Egypt [30]	RCT	N: 45 Sex: female Age: 56.4 ± 2.2 years	Flexible ruler Baseline kyphosis value: 62.2	D: 12 weeks F: 2 times per week I: NR Ti: 20–60 min T: combined diaphragmatic with corrective exercise	D: 12 weeks F: 2 times per week I: NR Ti: 20–60 min T: corrective exercise	TKA DE VAS QoL CVA	Both groups showed significant improvements post-intervention (*p* < 0.05), with no significant between-group differences, except for diaphragmatic excursion, which improved more in the combined diaphragmatic breathing and corrective exercise group (*p* < 0.05).
Elabd et al., (2025) Egypt [17]	RCT	N: 118 Sex: female Age: 13.7 ± 0.9 years	Cobb’s method Baseline kyphosis value: 50.7	D: 12 weeks F: 3 times per week I: NR Ti: 60 min T: combined diaphragmatic technique with corrective exercise	D: 12 weeks F: 3 times per week I: 70–80% of the maximum heart rate T: 60 min T: corrective exercise	TKA VC FVC MVV	The intervention combining corrective exercises and diaphragmatic technique led to greater improvements than exercises alone. TKA decreased by 8.14° vs. 5.92° (*p* < 0.003), with higher pulmonary gains in VC (0.94 vs. 0.44 L), FVC (1.02 vs. 0.67 L), and MVV (11.27 vs. 5.66 L/min, *p* < 0.001).
Afra et al., (2025) Iran [31]	Non- RCT	N: 45 Sex: male Age: 13.0 ± 0.8 years	Kyphometer Cobb angle: 48.7	D: 8 weeks F: 4 times per week I: NR Ti: 60 min T: combined breathing with corrective exercise	D: 8 weeks F: 4 times per week I: NR Ti: 60 min T: corrective exercise	TKA AF	The combined exercise group showed a significant reduction in kyphotic angle compared with the control group (*p* < 0.001). For aerobic performance, VO2peak increased significantly more in the combined exercise group than in the corrective group (η^2^ = 0.402).

Abbreviations: RCT: randomized controlled trial; N: number; NR: not reported; D: duration; F: frequency; I: intensity; Ti: time; T: type; TKA: thoracic kyphosis angle; CVA: craniovertebral angle; DE: diaphragmatic excursion; VAS: visual analog scale; QoL: quality of life; FVC: forced vital capacity; MVV: maximum voluntary ventilation; VC: vital capacity; CIE: cardioinspiratory endurance; AF: aerobic performance.

**Table 4:** Leave-one-out sensitivity analysis for thoracic kyphosis angle.

Study Omitted	Estimate	95%CI	*p*-Value
Haghighi et al. (2020) [32]	0.658	0.193 to 1.122	0.006
Munawwarah et al. (2023) [18]	0.692	0.230 to 1.153	0.003
Rashed et al. (2024) [30]	0.813	0.508 to 1.118	0.000
Elabd et al. (2025) [17]	0.625	0.088 to 1.162	0.023
Afra et al. (2025) [31]	0.557	0.230 to 0.885	0.001

**Table 5:** Summary of findings (GRADE): Movement-based exercises combined with respiratory techniques.

Outcome	Number of Studies (Design)	Effect Estimate (Hedges’ g, 95% CI)	Risk of Bias	Inconsistency	Indirectness	Imprecision	Publication Bias	Overall Certainty
TKA	5 (3 RCTs, 2 non-RCTs)	0.667 (0.395 to 0.940)	⊕⊕⊕	⊕⊕⊕	⊕⊕⊕	⊕⊕⊕	⊕⊕⊕	⨁⨁⨁◯ Moderate

Descriptions: CI: confidence interval; GRADE: Grading of Recommendations, Assessment, Development, and Evaluation; RCT: randomized controlled trial; TKA: thoracic kyphosis angle. In the GRADE system, the symbol ⊕ represents one level of evidence certainty, while ◯ indicates one level of downgrading. The total number of ⊕ symbols reflects the final certainty rating: ⊕⊕⊕ (high), ⊕⊕⊕◯ (moderate).

## Data Availability

The data presented in this study are available upon request from the corresponding author.

## Supplementary Materials

The following supporting information can be downloaded at https://www.mdpi.com/article/10.3390/healthcare14152384/s1: Table S1: PRISMA checklist; Table S2: Reasons for exclusion of studies.

## Abbreviations

The following abbreviations are used in this manuscript:

THK	Thoracic Hyperkyphosis
TKA	Thoracic Kyphosis Angle
RCT	Randomized Controlled Trial
