# Supplementary material for: The Effects of Movement-Based Exercises Combined with Respiratory Techniques on the Thoracic Kyphosis Angle in Individuals with Thoracic Hyperkyphosis: A Systematic Review and Meta-Analysis
Source: Healthcare (Basel). 2026 Aug 4;14(15):2384. doi: 10.3390/healthcare14152384 (PMC13465493; doi:10.3390/healthcare14152384)
Supplement: Supplementary file 1 [file healthcare-14-02384-s001.zip › Supplementary Table S2-Reasons for exclusion of studies.pdf]

Supplementary Table S2-Reasons for exclusion of studies

Supporting Information: Excluded studies with reason (n = 36).

Population	Exclusion reason
1. Park TS, Kim SH, Shin MJ. Cumulative Effects of Concurrent Inspiratory Muscle Training and Postural Correction Bands on Older Individuals Community Dwellers: A Randomized Study. Yonsei Medical Journal. 2024 Jun 5;65(8):472.	Not-kyphotic
2. Babina R, Mohanty PP, Pattnaik M. Effect of thoracic mobilization on respiratory parameters in chronic non-specific low back pain: A randomized controlled trial. Journal of Back and Musculoskeletal Rehabilitation. 2016 Aug;29(3):587-95.	Non-kyphotic
3. Banfi P, Redolfi S, Robert D. Home treatment of infection-related acute respiratory failure in kyphoscoliotic patients on long-term mechanical ventilation. Respiratory care. 2007 Jun 1;52(6):713-9.	Participants with kyphoscoliosis
4. Berdishevsky H. Outcome of intensive outpatient rehabilitation and bracing in an adult patient with Scheuermann's disease evaluated by radiologic imaging—a case report. Scoliosis and spinal disorders. 2016 Oct 14;11(Suppl 2):40.	Pathological kyphosis
5. Cejudo P, López-Márquez I, López-Campos JL, Márquez E, de la Vega F, Barrot E, Ortega F. Exercise training in patients with chronic respiratory failure due to kyphoscoliosis: a randomized controlled trial. Respiratory Care. 2014 Mar 1;59(3):375-82.	Participants with kyphoscoliosis
6. Gopaladhas S, Panigrah A, Chinnavan E, Ragupath R. Effectiveness of resisted abdominal exercise versus resisted diaphragmatic breathing exercise on cardio vascular endurance in sports men. International Journal of Medical Research & Health Sciences. 2014;3(4):785-9.	Not-kyphotic
7. López-de-Uralde-Villanueva I, Candelas-Fernández P, de-Diego-Cano B, Mínguez-Calzada O, Del Corral T. The effectiveness of combining inspiratory muscle training with manual therapy and a therapeutic exercise program on maximum inspiratory pressure in adults with asthma: a randomized clinical trial. Clinical rehabilitation. 2018 Jun;32(6):752-65.	Not-kyphotic
8. Radziejowska M, Malinowska A, Radziejowski P. Influence of breathing exercises using method of Body Flex by Greer Childers on to the selected somatic traits of women with overweight and obesity. Pedagogics, psychology, medical-biological problems of physical training and sports. 2014 Mar 28;18(3):57-63.	Not-kyphotic
9. Menadue C, Alison JA, Piper AJ, Wong KK, Hollier C, Ellis ER. High-and low-level pressure support during walking in people with severe kyphoscoliosis. European Respiratory Journal. 2010 Jul 31;36(2):370-8.	Participants with kyphoscoliosis
10. Park TS, Kim SH, Shin MJ. Cumulative Effects of Concurrent Inspiratory Muscle Training and Postural Correction Bands on Older Individuals Community Dwellers: A Randomized Study. Yonsei Medical Journal. 2024 Jun 5;65(8):472.	Not-kyphotic
11. Tsai CS, Li HT, Yang WL, Jhan YT. The effect of craniocervical flexion and neck endurance exercises plus pulmonary rehabilitation on pulmonary function in spinal cord injury: a pilot single-blinded randomised controlled trial. Spinal Cord Series and Cases. 2024 Apr 23;10(1):27.	Not-kyphotic
12. Dajah SA, Muthusamy H. Effects of back and respiratory muscle exercises on posture and respiratory function in elderly patients with osteoporosis. International Journal of therapy and rehabilitation. 2015 May 2;22(5):233-7.	Not-kyphotic
13. Vandevenne A, Ducolone A. Tolerance test to an inspiratory resistive load and resistive training of respiratory muscles. Preliminary critical study in kyphoscoliotic patients. Revue de Pneumologie Clinique. 1988 Jan 1;44(2):68-73.	Participants with kyphoscoliosis
14. Fuschillo S, De Felice A, Gaudiosi C, Balzano G. Nocturnal mechanical ventilation improves exercise capacity in kyphoscoliotic patients with respiratory impairment. Monaldi archives for chest disease= Archivio Monaldi per le malattie del torace. 2003 Oct 1;59(4):281-6.	Participants with kyphoscoliosis
15. Lisboa C, Moreno R, Pertuzé J, Cruz E. Inspiratory muscle training in patients with kyphoscoliosis. Revista Medica de Chile. 1987 Jun 1;115(6):505-11.	Participants with kyphoscoliosis
16. Vandevenne A, Ducolone A. Test de tolérance à une charge résistive inspiratoire et entraînement résistif des muscles respiratoires. Etude critique préliminaire chez 5 sujets cyphoscoliotiques [Tolerance test to an inspiratory resistive load and resistive training of respiratory muscles.	Participants with kyphoscoliosis

Preliminary critical study in kyphoscoliotic patients]. Rev Pneumol Clin. 1988;44(2):68-73. French. PMID: 3406619.	
17. Liu L, Lv J, Piao W, Liu X, Li S, Lu X. Research on the influence of yogalates comprehensive rehabilitation training on postoperative recovery of breast cancer patients. EXPLORE. 2023 Sep 22.	Not-kyphotic
18. Uçar Ü, Fırat SÇ, Ozdemir T, Lüleci E. Short-Term Exercise Improves Not Only Muscle Strength But Also Lung Capacity, Endurance, and Quality of Life in Postmenopausal Vertebral Osteoporosis. Archives of Rheumatology. 2025 Jun 23;40(2):211.	Not-kyphotic
**Intervention**	
1. Senthil P, Sudhakar S, Radhakrishnan R, Jeyakumar S. Efficacy of corrective exercise strategy in subjects with hyperkyphosis. Journal of back and musculoskeletal rehabilitation. 2017 Nov;30(6):1285-9.	Non-respiratory intervention
2. Hettchen M, Willert S, von Stengel S, Kohl M, Kemmler W. Effects of the "Spinomed active" orthosis on chronic back pain in kyphotic women with osteoporotic vertebral fractures three months and older: A randomized controlled study. Frontiers in Pain Research. 2022 Dec 21;3:1038269.	Non-respiratory intervention
3. Ghanbarzadeh M, Mehdipour A. Study and influence of exercise program on respiratory function of adults with kyphosis. Acta Bioeng Biomech. 2009 Jan 1;11(1):11-7	Non-respiratory intervention
4. KARDES, K., ALPOZGEN, A. Z. & AKGUL, T. 2026. Effect of video-based game therapy on activation of scapular muscles in children with thoracic hyperkyphosis. PM&R.	Non-respiratory intervention
**Comparison**	
1. Jang HJ, Kim MJ, Kim SY. Effect of thorax correction exercises on flexed posture and chest function in older women with age-related hyperkyphosis. Journal of Physical Therapy Science. 2015;27(4):1161-4.	Absence of a control group
2. Dajah SA, Muthusamy H. Effects of back and respiratory muscle exercises on posture and respiratory function in elderly patients with osteoporosis. International Journal of therapy and rehabilitation. 2015 May 2;22(5):233-7.	Absence of a control group
3. Le Solliec T, Blache Y, Rogowski I. Effects of an 8-week multimodal program on thoracic posture, glenohumeral range of motion and serve performance in competitive young tennis players. Frontiers in Sports and Active Living. 2023 Mar 3;5:1128075.	Absence of a control group
4. Jang HJ, Hughes LC, Oh DW, Kim SY. Effects of corrective exercise for thoracic hyperkyphosis on posture, balance, and well-being in older women: a double-blind, group-matched design. Journal of geriatric physical therapy. 2019 Jul 1;42(3):E17-27.	Absence of a control group
5. Meamari H, Koushkie Jahromi M, Fallahi A, Sheikholeslami R. Influence of structural corrective and respiratory exercises on cardiorespiratory indices of male children afflicted with kyphosis. Archives of Rehabilitation. 2017 Jun 10;18(1):51-62.	The control group did not receive identical movement-based exercise
6. Sedaghati P, Derakhshan KF, Ahmadabadi S, Moghaddam SR. Effects of corrective and breathing exercises on respiratory function of older adults with a history of COVID-19 infection: a randomized controlled trial. BMC complementary medicine and therapies. 2023 Jun 16;23(1):199.	The control group did not receive identical movement-based exercise
7. Eftekhari E, Sheikhhoseini R, Salahzadeh Z, Dadfar M. Effects of telerehabilitation-based respiratory and corrective exercises among the elderly with thoracic hyper-kyphosis: a clinical trial. BMC geriatrics. 2024 Mar 6;24(1):234.	The control group did not receive identical movement-based exercise
8. Rezazadeh SZ, Azimkhani A, Alirezaei Noghondar F. The effect of eight weeks of combined NASM and breathing exercises on spine alignment, respiratory capacity, and quality of life in the elderly with hyperkyphosis: A randomized clinical trial.	The control group did not receive identical movement-based exercise
9. HASHEMINEZHAD, S., DARYABOR, A., SAFIKHANI, H. & GHORBANI, N. 2026. The Effects of Corrective Exercises and Respiratory Biofeedback on the Kyphosis Angle and Electrical Activity of Trapezius Muscles in Male Adolescents With Kyphosis: A Randomized Controlled Trial. HEALTH SCIENCE REPORTS, 9.	The control group did not receive identical movement-based exercise

10. JANNAT, M. G., DAMIRCHI, A. & MAJELAN, A. S. 2025. The Effect of Corrective and Aerobic Exercises on General Health Index, Body Composition of Women with Postural Hyperkyphosis. Sadra Medical Sciences Journal, 13, 343-355.	The control group did not receive identical movement-based exercise
**Outcome**	
1. BRILLE D, HATZFELD C, LEJEUNE F. The treatment of alveolar hypoventilation in cardiorespiratory failure in kyphotic patients. Journal francais de medecine et chirurgie thoraciques. 1963;17:181-90.	Absence of an outcome related to kyphosis
**Study type**	
1. Tsekoura M, Katsoulaki M, Kastrinis A, Nomikou E, Fousekis K, Tsepis E, Billis E. The effects of exercise in older adults with hyperkyphotic posture. InWorldwide Congress on "Genetics, Geriatrics and Neurodegenerative Diseases Research" 2022 Oct 20 (pp. 501-506). Cham: Springer International Publishing.	Non-original study
2. Bastow J. Some orthopaedic procedures employed in the treatment of arthritis. Proc R Soc Med. 1941;34(11):801–804	Non-original study
3. Yoo WG. Effects of combined chest expansion and breathing exercises in a patient with sternal pain. Journal of Physical Therapy Science. 2017;29(9):1706-7.	Case report
